# Nipah Virus: An Overview of the Current Status of Diagnostics and Their Role in Preparedness in Endemic Countries

**DOI:** 10.3390/v15102062

**Published:** 2023-10-07

**Authors:** Anna Rosa Garbuglia, Daniele Lapa, Silvia Pauciullo, Hervé Raoul, Delphine Pannetier

**Affiliations:** 1 Laboratory of Virology, National Institute for Infectious Diseases “Lazzaro Spallanzani” (IRCCS), 00149 Rome, Italy; daniele.lapa@inmi.it (D.L.); silvia.pauciullo@inmi.it (S.P.); 2 French National Agency for Research on AIDS—Emerging Infectious Diseases (ANRS MIE), Maladies Infectieuses Émergentes, 75015 Paris, France; herve.raoul@inserm.fr; 3 Institut National de la Santé et de la Recherche Médicale, Jean Mérieux BSL4 Laboratory, 69002 Lyon, France; delphine.pannetier@inserm.fr

**Keywords:** Nipah virus, zoonosis, One Health, molecular diagnosis, infection

## Abstract

Nipah virus (NiV) is a paramyxovirus responsible for a high mortality rate zoonosis. As a result, it has been included in the list of Blueprint priority pathogens. Bats are the main reservoirs of the virus, and different clinical courses have been described in humans. The Bangladesh strain (NiV-B) is often associated with severe respiratory disease, whereas the Malaysian strain (NiV-M) is often associated with severe encephalitis. An early diagnosis of NiV infection is crucial to limit the outbreak and to provide appropriate care to the patient. Due to high specificity and sensitivity, qRT-PCR is currently considered to be the optimum method in acute NiV infection assessment. Nasal swabs, cerebrospinal fluid, urine, and blood are used for RT-PCR testing. N gene represents the main target used in molecular assays. Different sensitivities have been observed depending on the platform used: real-time PCR showed a sensitivity of about 103 equivalent copies/reaction, SYBRGREEN technology’s sensitivity was about 20 equivalent copies/reaction, and in multiple pathogen card arrays, the lowest limit of detection (LOD) was estimated to be 54 equivalent copies/reaction. An international standard for NiV is yet to be established, making it difficult to compare the sensitivity of the different methods. Serological assays are for the most part used in seroprevalence studies owing to their lower sensitivity in acute infection. Due to the high epidemic and pandemic potential of this virus, the diagnosis of NiV should be included in a more global One Health approach to improve surveillance and preparedness for the benefit of public health. Some steps need to be conducted in the diagnostic field in order to become more efficient in epidemic management, such as development of point-of-care (PoC) assays for the rapid diagnosis of NiV.

## 1. General Aspects

**Figure 1:**
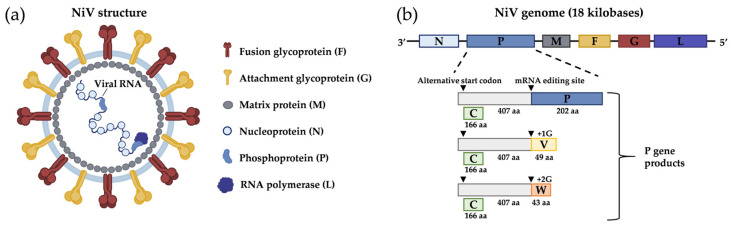
Nipah virus (NiV) structure and genome organization. (**a**) Enveloped NiV virion comprises different structural proteins: nucleoprotein (N), phosphoprotein (P), matrix protein (M), fusion glycoprotein (F), and attachment glycoprotein (G). Viral RNA polymerase (L) and N proteins are associated with the viral genome (negative-sense single-strand RNA). They are created by BioRender. (**b**) Schematic representation of NiV genome organization. Genes encoding N, P, M, F, G, and L proteins are shown. P gene encodes accessory proteins using an alternative start codon (C protein) or using mRNA editing (V and W proteins).

Nipah virus (NiV) infection is a viral disease that has emerged in Southeast Asia and is caused by a negative single-stranded RNA virus of 18,000 nucleotides in length, which belongs to the Paramyxoviridae family and to the *Henipavirus* genus. This genus also includes other species that can infect humans such as Ghanaian bat virus, Mojiang virus, and Hendra virus (HeV) [1]. First detected in the 1998–1999 outbreaks in Malaysia and Singapore, NiV is responsible for a zoonosis with mainly severe respiratory and neurological clinical manifestations in humans and regular outbreaks in Bangladesh, India, or the Philippines, and the morbidity and the mortality rate are related to the viral strain [2]. The high fatality rates in humans associated with NiV of up to 70% [3] has led to their classification as risk-group 4 pathogens, restricting work on these viruses to Biosafety Level 4 (BSL-4) facilities. The BSL categories take into account the lethality of the disease and the availability of preventive and therapeutic treatments, which do not currently exist in the case of NiV or HeV. NiV and HeV genomes share about 80% nucleotide identity [4], so diagnostics tests can be cross-reactive between these two viruses depending on the RNA sequence targeted. Similar to all paramyxoviruses, the genome codes for the following proteins: nucleocapsid protein (N), phosphoprotein (P), matrix protein (M), glycoprotein F (F), glycoprotein G (G), and RNA polymerase, which represents the large protein (L). The non-structural proteins C, V, and W, which play a key role in the pathogenicity of NiV, are encoded by the P gene [5]. The RNA genome is associated with the viral proteins of the replicative complex, which include nucleoprotein, phosphoprotein, and the polymerase L enclosed by a lipid bilayer envelope containing the attachment protein G and the fusion F protein [6]. The receptor of NiV is ephrin-B2, present in the endothelial cells and neurons [7,8,9]. The NiV-G and -F proteins are necessary for the binding and fusion to the host cells and for budding [10,11] (Figure 1).

## 2. Epidemiology

The first human cases of NiV were observed in Malaysia in 1998–1999, near Sungai Nipah (Nipah River village). In this outbreak, close contacts with pigs, pig excreta, or fruit bats were shown to be the main risk factors for disease transmission [12,13].

### 2.1. Fruit Bats

*Pteropus* bats were identified to be a reservoir of NiV infection in Malaysia [14]. NiV neutralizing antibodies were detected in blood, and NiV RNA was detected in urine, saliva, serum, and different organs of *Pteropus* bats in many regions of Asia, and also in countries where no human infections had been described [15,16,17]. The *Pteropus* bats are widely distributed in Southeast Asia, the Indian Ocean, Oceania, and Sub-Saharan Africa [18,19]. In India, in May 2018, 18 NiV human infections were observed in the State of Kerala with patients showing acute respiratory syndrome and encephalitis [20,21]. The human NiV strains showed 99.7–100% sequence homology with bat NiV strains, suggesting that bats were the source of outbreak [22]. These fruit bats could infect humans or pigs through the consumption of bat-bitten fruits amplifying the virus diffusion.

### 2.2. Pigs

It is commonly accepted that pigs contract NiV infection by eating food contaminated with urine or saliva from bats and can then infect humans. The main symptom in pigs infected with NiV is a respiratory disease with about 5% lethality. Pig farmers and abattoir workers who are in direct contact with infected animals are the main group at risk for NiV infection [13,23]. For example, an outbreak among slaughterhouse workers who looked after pigs from Malaysia occurred in Singapore in 1999. The epidemic was limited with a ban on the import of pigs from Malaysia [24]. NiV was isolated from the nose and oropharynx swabs of pigs, which are animals that can act as intermediate hosts [6,12,25,26].

### 2.3. Other Hosts

Serological studies have demonstrated that other animals such as cattle, horses, dogs, cats, or goats can also be exposed to NiV and can develop specific antibodies against NiV, although transmission to humans in this way is yet to be reported [6,23,27].

## 3. Modes of Transmission

After the Malaysia outbreak in 1998–1999, later outbreaks of NiV confirmed that the main routes of virus transmission to humans are direct contact with the respiratory secretions or body fluids of infected animals such as bats and pigs or by the consumption of contaminated fruit palm or its derivatives (sap and alcohol). Indeed, several human outbreaks originated from drinking contaminated raw palm sap or climbing the trees coated with contaminated excrement, urine, or saliva from fruit bats [28,29]. Human outbreaks can occur sporadically (as observed in India and The Philippines) or in more specific times of the year, such as in the winter season in Bangladesh during the date palm harvest [30]. In the 2014 Philippines outbreak, close contact with horses or horse meat consumption was reported in 10 of the 17 confirmed cases. In the same period, 10 horses died, 9 of which were reported to have neurological disorders, although no test for NiV was performed on the horse samples [31]. In this study, fruit bats were considered as the most probable source of infection for horses [31].

Human-to-human transmission has also been reported [12,32,33]. For example, in India in 2007, an outbreak originated from one person who contracted the disease due the consumption of alcohol obtained from date palm. The infection was transmitted to other members of the family and to the One Health worker who collected blood and performed a tomography scan of the brain of the initial disease case, suggesting that close contacts are required to transmit the disease [34]. Indeed, contact with the body fluids of NiV positive patients increases the risk of virus transmission [35]. The Bangladesh strain of NiV is usually associated with human-to-human transmission, with no intermediary host and with a case fatality rate of 75% [12,36].

## 4. Symptoms and Pathogenesis in Humans

In humans, the infection mainly occurs via the oronasal site, and different incubation periods are reported in the literature, mainly from 1 to 2 weeks, but ranging from 4 to 21 days [37,38,39,40]. According to the World Health Organization (WHO), the period of incubation for NiV could vary from 4 to 45 days, but mainly from 4 to 14 days [41]. In the outbreak in Malaysia and Singapore in 1998–1999, 3.3 days was the meantime between fever onset and hospitalization, whereas death occurred after 9.5 days [42].

Different clinical courses have been described, and they vary according to the NiV strain. Differences in symptoms and lethality rate have been observed between the Malaysian (NiV-M) and Bangladesh strains (NiV-B). NiV-B is often associated with severe respiratory disease, whereas NiV-M is more often associated with severe encephalitis. Furthermore, in 2018, during the outbreak of Kerala caused by NiV-B, 83% of patients showed respiratory distress syndrome (ARDS) [36,38]. The course of the disease also depends on the strain: the mean disease duration from symptom onset to death was 16 days in Malaysia and only 4–6 days in Bangladesh and India [43,44]. The variability of interval time between symptom onset and death, which has been observed in different outbreaks, is probably linked to the different virulence of the strains.

Generally, the beginning of NiV disease is characterized by flu-like symptoms, with fever, myalgia, cough, vomiting, and headaches [12,39,45].

Respiratory complications characterized by cold, respiratory distress, shortness of breath, and atypical pneumonia can be observed. The poor prognosis is linked to age, thrombocytopenia, or the presence of other comorbidities [39]. In the late stage, NiV-M infection can also reach the Central Nervous System (CNS) with several degrees of encephalopathy. Neurological symptoms include convulsions, altered functionality of cerebellum, and reduction in consciousness. Different levels of consciousness reductions have been reported, 50% in Malaysia versus 90% in Bangladesh, with NiV-B inducing more severe respiratory troubles than NiV-M [4]. Problems of mental disorientation can also appear [46]. The most common symptoms of encephalitis that could arise after a week are hypotonia, segmental myoclonus, areflexia, limb weakness, and gaze palsy. Coma and death can then happen within a few days. Fatigue, neurological deficits, and depression can persist in about 20% of patients who recover from acute infection [47], and some cases of relapsing or late-onset encephalitis have been described [48]. Furthermore, latent infections have been described after acute infection with a persistence that can last months or even years [49].

Subclinical infections have also been described in NiV epidemics. The rate of asymptomatic infections among confirmed cases varies according to the outbreak, ranging from 8 to 17% in Malaysia to more than 45% in Singapore [4,13,24,50,51]. Asymptomatic infections were absent or rarely observed with NiV-B in Bangladesh and India [52,53].

NiV infection leads to local changes in blood vessels with the appearance of vasculitis in the small vessels in humans. Brain parenchyma often presents necrotic plaques and sometimes syncytia of multinucleated giant endothelial cells. Moreover, the infection spreads to major organs [6,32,45,54].

## 5. Diagnostics

### 5.1. Molecular Diagnostics

In suspected cases, an early diagnosis of NiV infection is crucial to limit the outbreak and to provide appropriate care for the patient. NiV infection can be confirmed in several ways using direct detection methods, such as virus isolation, immunohistochemistry or immunofluorescence assays, nucleic acid amplification, or sequencing, but also using indirect detection methods of anti-NiV IgM or IgG antibodies, such as enzyme-linked immunosorbent assays (ELISA) or virus neutralization tests. Due to high specificity and sensitivity, qRT-PCR is currently considered as the first-choice method for the diagnosis of acute NiV infection, and it can reliably diagnose NiV infection within a few hours. As described above, NiV has a period of incubation which mainly ranges from 4 to 14 days, even though it can be longer [55]. It is yet to be established whether or not patients can transmit the virus during the incubation period, but such transmission has been demonstrated in pigs [56]. Nasal swabs, cerebrospinal fluid, urine, and blood are usually used in RT-PCR tests [57].

Originally, heminested or nested RT-PCR were the main methods used to detect NiV RNA. Real-time RT-PCR was set up later and, compared to conventional RT-PCR, provides a better sensitivity, an ease of use, and a reduced risk of contamination, so it has become the gold standard diagnostic method. Real-time PCR is also indicated by the CDC as the reference method to be used for the diagnosis of acute infection [58].

Different kinds of RT-PCR have been set up for NiV detection, most of them being in-house assays (Table 1). Some RT-PCRs are able to detect several paramyxoviruses including NiV. For example, a nested RT-PCR described by Tong [59] is still used for known or new paramyxovirus identification. It consists of a broad range RT-PCR that uses a conserved region of L gene as a target. The sensitivity ranged between 500 and 1000 copies of template RNA [59]. Another duplex RT-PCR was set up for RNA detection from the urine of bats. This RT-PCR included an internal control (IC) consisting of an RNA plasmid containing a 1.2 kb fragment of Kanamycin gene. The primers for NiV were specific for a conserved region of N gene, as described previously [6]. The lower limit of detection was 0.37 pg/µL of total RNA. This method was also applicable to other biological samples such as bat’s saliva and blood [60,61]. One of the most frequently used Real-Time PCR was developed by Guillaume [62] and is based on the amplification of a conserved region of the N gene. This assay has a linearity between 10^3^ and 10^9^ equivalent copies of NiV. It was set up with the NiV-M strain, but the authors did not report whether this sensitivity was confirmed for the NiV-B strain. This assay is specific for NiV and does not amplify Hendra nor measles virus genome. A comparison of TaqMan Real-Time and SYBR Green assay using primers designed in different regions of NiV virus (for N, M, L, and P genes) showed different sensitivities according to the Real-Time PCR platform [63]. Since it was not possible to set up a TaqMan Real-Time PCR assay for L gene, a heminested RT-PCR was carried out for this gene. In this study, one-step RT-PCR appeared to be more sensitive than two-step RT-PCR. Assays based on N and P genes showed the same sensitivity in SYBRGreen format, while P gene was found to be the most sensitive in TaqMan platform. The SYBR Green method can distinguish NiV and HeV on the basis of melting curve analysis. The TaqMan assays for P and N regions, N SYBR Green protocols, and L heminested RT-PCR showed a sensitivity of 20 equivalent genomes per reaction, while M gene TaqMan assay was associated with a sensitivity of 2000 equivalent genomes per reaction. In this case, the limited sensitivity of M gene RT-PCR could be linked to the use of degenerate primers in the assays. M and P gene primer pairs were found to be unsuitable for SYBR Green assays, because the potential primer dimers probably interfere with the performance of the assay [63].

N gene was also used as a target in a SYBR Green I-based qRT-PCR amplification assay used to analyze the kinetics of viral replication in vitro, with a sensitivity of about 100 pfu/µL [64]. For a precise molecular quantification of the viral burden, it is important to note that in most of the real-time assays, target sequences are present both in genomes and in mRNA transcripts. Thus, the viral burden estimation can be influenced by the amount of mRNA transcripts. For example, N transcripts are the most abundant, and L transcripts are the least abundant. A real-time approach to calculate the real burden of genomic NiV RNA excluding amplification of the viral mRNA was developed by Jensen [65]. This one-step RT-PCR targets only viral genomic RNA in the untranslated intergenic region separating the F and G viral proteins and does not amplify mRNA transcripts, making it also possible to detect both NiV-M or NiV-B isolates in the same sample. Detection is linear from 1 × 10^2^ to 1 × 10^9^ copies/mL with a coefficient of correlation of r^2^ = 0.998. Different LOD were reached with NiV-B and NiV-M strains with 1.63 × 10^4^ genomes/mL compared to 5.82 × 10^3^ genomes/mL, respectively. No cross reactivity was found with Hendra virus (HeV), Respiratory Syncytial Virus (RSV), Ebola virus (EBOV), or Lassa virus (LASV).

Moreover, in order to improve the rapidity of the diagnostics and to test different pathogens at the same time, multiple pathogen card arrays are often used. Different types of platforms exist on the market, and one of the main advantages of these cards is the relatively easy use and storage as well as the stability at 4 °C for two years with shipment at room temperature. For example, one TaqMan array card (TAC) based on N region is able to detect several pathogens simultaneously in cerebrospinal fluid: *Balamuthia mandrillaris* and *Acanthamoeba* (parasites), *Streptococcus pneumoniae*, *Haemophilus influenzae*, *Neisseria meningitidis*, *Mycoplasma pneumoniae*, *Mycobacterium tuberculosis*, and *Bartonella* (bacteria), and 13 viruses (parechovirus, dengue virus, Nipah virus, varicella-zoster virus, mumps virus, measles virus, lyssavirus, herpes simplex 1 and 2, Epstein Barr virus, enterovirus, cytomegalovirus, and chikungunya virus). The target sequence for NiV is the N gene, and the estimated LOD is 54 equivalent copies/well [66].

Another TAC is based on quantitative reverse transcription PCR (qRT-PCR) for the simultaneous detection of 15 viruses including NiV (Chikungunya, Crimean-Congo hemorrhagic fever (CCHF) virus, dengue, EBOV, Bundinbugyo virus, SUDV, hantaviruses (Hantaan and Seoul), Hepatitis E, MARV, Nipah virus, O’nyong-nyoung virus, Rift Valley fever virus, West Nile virus, and YFV), 8 bacteria (*Bartonella* spp., *Brucella* spp., *Coxiella Burneti*, *Leptospira* spp., *Rickettsia* spp., *Salmonella enterica* and *Salmonella enterica* serovar Typhy, and *Yersinya pestis*), and 3 protozoa (*Leishmania* spp., *Plasmodium* spp., and *Trypanosoma brucei*) of particular relevance to Sub-Saharan Africa. TAC exhibited an overall sensitivity of 88% and a specificity of 99%. The LOD for viral genomes is 10^4^ copies/mL in blood. No indication is available for cerebrospinal fluid samples. A critical aspect of this assay is the need of a nucleic extraction step that limits its use for NiV surveillance activities in remote areas and could also induce some biosafety problems [67].

Nevertheless, it can be difficult to perform such RT-PCR tests if the outbreak occurs in a remote area with limited facilities, especially for electrical power and working materials. In such cases, other types of PCR techniques could be implemented. For example, N gene is also the target for real-time reverse transcription-loop-mediated isothermal amplification (RT-LAMP), which is able to detect all NiV strains [68]. The results are obtained within 45 min, and the sensitivity is close to 100 pg of total M and B genotypes NiV pseudovirus RNA, corresponding to 10^7^ RNA copies. This is ten fold higher than the sensitivity obtained with a conventional N gene RT-PCR [69]. No cross reactivity was found with Influenza A virus and Hendra virus. This assay was able to detect NiV in different biological samples: urine, blood, feces, and throat swabs [68].

Recently, three rapid molecular diagnostic tests were developed. They are based on reverse transcription recombinase-based isothermal amplification coupled with lateral flow detection. For these three assays, a region of N gene is targeted and reaches an analytical sensitivity of 1000 copies/µL, corresponding to 100–200 RNA copies/reaction, for both NiV-B and NiV-M genotypes. These tests provide the results within 30 min without any extraction step allowing their use in low-resource settings [69].

Finally, NiV can also be detected in biological samples using a metagenomic approach based on high-throughput sequencing (HTS). These metagenomic approaches are used frequently nowadays and could become the new reference technique in the future. A combination of RNA baits specific for 35 epizootic and zoonotic viruses made it possible to enrich the samples from about 10- to 10,000-fold, reaching a considerable sensitivity. For NiV, this assay showed a sensitivity of 21 genomes/reaction [70].

**Table 1:** Molecular diagnostic assay of Nipah virus.

Test	Test Type	Virus Detected	Target Gene	Biological Matrix	Sensivity	Specificity	Author
Home made	RT-PCR broad range	Paramyxoviruses	L gene of NiV	Paramyxovirus viral strains (henipavirus, Morbilliviruses, respiroviruses, and Rubulaviruses)	10–100 copies/reaction	No cross reactivity with Influenza A and B viruses, Rhinoviruses, adenoviruses, coronaviruses 229E, and OC-43. *Chlamidia pneumoniae*, *Haemophilus influenzae*, *Streptococcus pneumoniae*, and *Mycoplasma pneumoniae*	Tong et al., 2008 [59]
Home made	Duplex RT-PCR	NiV	N gene of NiV	Urine, saliva, and blood	0.37 pg/µL	ND	Chua et al., 2000 [6]
Home made	Real-time RT-PCR with fluorescent reporter dye detected at each PCR cycle	NiV	N gene of NiV M strain	Viral stock	Whole blood: 10^3^ copies/mL	No cross-reactivity with measles virus was declared	Guillaume et al., 2004 [62]
Home made	Real-time RT-PCR with fluorescent reporter dye detected at each PCR cycle	NiV	N, M, P genes	In vitro transcribed RNA	20 copies/reaction	Whole blood: 100% [95.9–100].	Feldman et al., 2009 [63]
Home made	SYBR Green RT-PCR	NiV	N gene	Whole blood and urine	20 copies/reaction	Whole blood: 100% for Nipah virus	Feldman et al., 2009 [63]
Home made	Transcription-loop-mediated isothermal amplification (RT-LAMP)	NIV	N gene of NiV	Whole blood sample, fecal sample, throat swab sample, and urine sample	10^7^ copies/ reaction	No cross reactivity with HeV, Newcastle disease virus, Japanese encephalitis virus, and Influenza A virus.	Ma et al., 2019 [68]
Home made	SYBR Green RT-PCR	NiV	N gene of NiV	Viral replication in vitro	100 pfu/µL	ND	Chang et al., 2006 [64]
Home made	Real-time RT-PCR with fluorescent dye-labelled probes to detect PCR amplicons	NiV	Intergenic region separating the viral F and G protein coding regions	Serum, body fluid, and urine	LOD: NiV-B assay 1.63 × 10^4^ genomes/mL; NiV-M 5.82 × 10^3^ genomes/mL	No cross reactivity with HeV, RSV, EBOV, and LASV	Jensen et al., 2018 [65]
Home made	NGS	35 epizootic and zoonotic viruses	Full genome	Whole blood, serum, plasma, and urine	21 genomes/reaction	ND	Wylezich et al., 2021 [70]
EZ1 test (DOD)	Real-time TaqMan RT-PCR with fluorescent reporter dye detected at each PCR cycle	Several pathogens	N gene	Whole blood and plasma	Whole blood: 54 copies/well	100%; no cross-reactivity with other Viral Haemorrhagic Fever	Onyango et al., 2017 [66]
Home made	TaqMan array CARD	15 viruses, 8 bacteria and 3 protozoa			Blood samples 10^4^ copies/mL		Liu et al., 2016 [67]
Home made	Reverse transcription recombinase-based isothermal amplification coupled with lateral flow detection		N gene	Viral stock produced in vitro	10^3^ copies/µL (analytical sensitivity)	No cross reactivity with Hendra virus	Pollak et al., 2023 [69]

### 5.2. Serological Diagnosis

**Figure 2:**
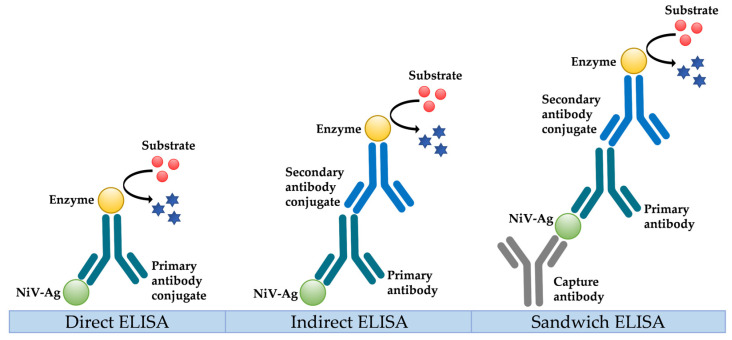
Different types of enzyme-linked immunosorbent assay (ELISA).

RT-PCR is the best option to diagnose NiV in humans, especially during the acute phase of infection, but indirect antibody detection methods may be used to diagnose later NiV infections or as a complementary diagnostic method. IgG and IgM are indeed key markers for Nipah seroprevalence studies, but data on kinetics or persistence during human convalescence are limited. ELISA-based serological tests can be used to detect IgM and IgG antibodies in the serum and cerebrospinal fluid (CSF), and it seems that for most patients, the IgM and IgG antibodies appear in the serum during the first week after onset (Figure 2) [71]. IgG antibodies seem to persist up to 8 months in symptomatic patients, while IgM antibodies seem to persist from 3 to 7 months. According to Ramasundram et al., IgM positivity is observed on the first day after the appearance of symptoms in 50% of patients and reaches 100% after three days, while IgG are detected two days after the onset of symptoms in 31% of patients, with 100% positivity reached by day 17 [72]. In a small study performed in India [73], contact cases of NiV-positive patients were tested regularly for a number of months to detect IgM and IgG antibodies. Antibodies were detected among three symptomatic and two asymptomatic contact cases. For symptomatic patients, IgM was detectable from the 5th to 27th days following disease onset, while IgG can persist for more than one year. Comparable IgM and IgG immune responses against NiV infection were observed in the presence or the absence of clinical symptoms.

Due to their more complicated use compared to RT-PCR, need of specific reagents, and longer implementation time, serological tests are more commonly used in epidemiological studies and surveillance activities with no ongoing NiV acute infections. Several tests have thus been implemented over the years to detect NiV IgG and IgM. These tests can generally differentiate NiV from HeV [74,75,76,77].

Initially, ELISA investigations used a gamma-irradiated NiV antigen [78,79]. However, in this assay, the antigen derived from NiV-infected cells was difficult to standardize due to the variability of different culture parameters such as virus strains, cell line, or multiplicity of virus infection, and an antigen produced in different culture conditions could have different antigenic properties causing potential troubleshooting in the interpretation of the tests. The main advantage of these tests is their ability to detect a very broad spectrum of human antibodies directed against the whole virus and not only restricted to several proteins. On the other hand, more recent serological tests using NiV recombinant proteins allow a greater standardization but cannot detect a large spectrum of antibodies directed against the whole virus.

N recombinant protein is most frequently used in ELISA assays because of its immunodominance and conservation among different NiV strains. This structural protein is the most abundant produced during the infection by NiV, and amino acidic sequences are similar in humans and animal reservoirs [6,80]. NiV-N recombinant protein has been often expressed in *E. coli*. Yu et al. [81] developed an ELISA with the NiV-N recombinant protein that showed a sensitivity and specificity of 91.7% and 91.8% respectively, using human sera and the CDC IgM ELISA assay as a reference test. The NiV antibody detection in swine sera showed a 100% concordance with the CDC IgG ELISA assay. These findings confirmed that N protein is a highly immunogenic protein that must be considered as an excellent target for serodiagnosis assays.

In another assay, the NiV-N protein expressed in *E. coli* was tested in 1709 swine serum samples and showed a sensitivity and specificity of 100% and 98.7%, respectively [82].

In addition to direct ELISA assays, antigen capture ELISA could also be implemented to detect HeV and NiV antibodies. The anti-N antibody 1a11c1 captures proteins from HeV and both NiV-M and NiV-B strains with a high sensitivity (LOD 400 pfu/well) and can detect NiV antigen from a frozen pig lung specimen. No cross-reactivity was observed with Marburg hemorrhagic fever (MHF) virus. The 1a11c1 antibody detects the NiV-M SPB199901924 Malaysia strain [83,84,85] but a low background signal was observed for uninfected Vero cell lysate, Lassa virus, Marburg, and Crimean-Congo hemorrhagic fever (CCHF), indicating a lack of specificity of assays performed with this antibody. Indeed, a problem linked to NiV-N protein expression is the non-specific binding due to residual bacterial *E. coli* proteins, which are not totally removed during the purification process [86]. This aspect has been addressed by Chen [87] by calculating the background binding due to non-specific interactions using incubation of the sera with excess free antigen to block specific binding. The sample was considered positive only when its total reactivity signal was higher than a pre-determined cut-off value, and the ratio of the total reactivity to the background signal was A450 R sample reactivity/A450 B background reactivity > 2.0. This method eliminates 35% of reactive but non-specific sera, and reached a specificity of 95.8%, which is no higher than those reported by other ELISA assays. 

Another ELISA, named solid-phase blocking ELISA, was developed to detect anti-NiV antibodies [88]. The ELISA plates were coated with NiV virus cell infected lysate, and the serum incubated with solid phase was washed after one hour. Anti-NiV monoclonal antibodies (mAb) conjugated to peroxidase were then added to detect the remaining free antigen. A sample was considered positive when the inhibition due to mAb reaction against NiV antigen was over 20%. The relative sensitivity and specificity against the serum neutralization test were >70% and >95%, respectively, with human sera. Considering its lower sensitivity compared to other ELISAs, this test should only be used for anti-NiV screening. Luminex platform is also used for antibody detection. This type of assay uses very small sample volumes, can be used in multiplex, and is much cheaper than traditional ELISA assays and therefore is of relevance to low- and middle-income settings [89,90]. Serum neutralization assay as a confirmatory test should be then performed to confirm the results for doubtful values and to avoid false positive results. This assay was employed to test pig and bat sera but could also be used for other animal species.

### 5.3. Neutralization Assays

Considering its high specificity, serum neutralization testing is today considered as the reference standard for the confirmatory diagnosis of NiV by the World organization for Animal Health (OIE). Moreover, these tests are useful to check the protective immunity of a serum that is correlated to the level of neutralizing antibodies. First neutralization assays were initially developed with live virus. The sera are serially diluted and incubated with a well-defined number of viral particles, before being added to Vero cells. After 24 h, the syncytia specific of NiV infection can be observed [91]. Such an assay with infectious virus has two main limitations as it must be conducted in a BSL4 laboratory, and the plaque reduction observed could be linked to serum cytotoxicity instead of being a direct effect produced by the virus, even though the serial dilutions used during the test limit this risk.

One alternative to standard plaque reduction neutralization tests (PRNT) is a neutralization assay based on pseudovirions that can be used to detect antibodies against viral surface glycoproteins. Since pseudovirions are able to produce non-infectious viruses, they do not require BSL4 facilities and could be easy to implement. The envelope attachment (G) and fusion (F) glycoproteins can be expressed in different systems: Moloney murine leukemia virus (MuLV), vesicular stomatitis virus (VSV), or human immunodeficiency virus lacking envelope protein [92,93,94]. The reporter gene can be the green fluorescent protein (GFP) or luciferase [95,96]. All these assays based on pseudovirions are quantitative, but some limitations exist as the sensitivity of each assay has not always been compared with the sensitivity obtained using PNRT, which is considered as the gold-standard neutralization assay. Some aspecific positivity has also been observed. For example, a study using pig vaccinated serum compared the results obtained with PRNT assays and tests with pseudovirions produced by VSV expressing the F and G proteins of NiV as target antigens (pVSV-NiV-F/G) [96]. In this study, there is a good correlation in the results obtained with the two assays, except to detect low levels of antibodies where PRNT results were better than pseudovirion test results. The specificity was high (94–100%). Therefore, at least for the moment, neutralization assays performed with pseudovirions cannot totally replace the assays with live viruses.

### 5.4. Virus Isolation

Before RT-PCR, virus isolation was the reference method to confirm the diagnostics of NiV infection, because it could characterize the strain precisely. Indeed, through viral isolation, one can obtain enough material to sequence the whole genome or to set up neutralization assays. Nevertheless, viral isolation requires BSL4 facilities, it is time-consuming and less sensitive than RT-PCR because it is not always possible to isolate a virus from a sample. It is therefore no longer used for primary diagnosis but only for confirmatory diagnosis and to work further with the isolated viral strain. NiV can be isolated from throat swabs, urine, CSF, nasal swab samples, and brain and lung tissues using the Vero E6 cell line. A cytopathic effect usually appears within 3 days. It is characterized by the formation of syncytia that may contain 20 or more nuclei. Subsequently, syncytia detach from the substrate leaving holes in the monolayer surface [78]. This technique can be used to differentiate NiV from HeV. Indeed, for NiV, the nuclei and nucleocapsid are localized in the periphery of the infected cells, while in the case of HeV, both structures tend to localize centrally [97].

Moreover, different studies report a correlation between a positive virus isolation in the CSF and a high lethality in patients [98].

## 6. One Health Concept for Preparedness Applied to Nipah Virus

NiV outbreaks occur regularly in India and Southeast Asia. Surveillance of NiV circulation in endemic countries is therefore crucial for pandemic preparedness but must be performed with adapted diagnostic tests and using the One Health approach.

As indicated on the WHO website, the One Health concept is an integrated, unifying approach to balance and optimize the health of people and animals and the environment [99]. This approach mobilizes multiple sectors, disciplines, and communities at varying levels of society to work together to prevent, predict, detect, and respond to global health threats such as pandemics. One Health involves the public health, veterinary health, and environmental approach and thus can be implemented to improve the surveillance of this pathogen and to minimize the risk of large NiV outbreaks. Indeed, due to the following characteristics, NiV represents a large threat for both animal and human health due to the reservoir of NiV is *Pteropodidae* bats, which are widely distributed in Southeast Asia in densely populated areas; direct transmission to humans could occur via bats or domesticated animals; human-to-human transmission is possible; emergence is often described in densely and connected areas; the mortality rate is high; and there is no effective vaccine or treatment. All these reasons contribute to making NiV a high-risk pathogen priority for the WHO, and using the One Health method seems to be of great importance for NiV preparedness. Since the SARS-CoV-2 pandemic, the One Health approach for infectious diseases has indeed become more significant because prevention and preparedness is the key to better understand the risks of emergence and to limit pandemics.

Due to the mutual dependence between humans and animals, wild animal trade or hunting, deforestation, climate change, intensive agriculture or unlimited urbanization increase contacts between humans and animals and thus also the risk of viral emergence. For example, very recently in Vietnam, the possibility of the emergence of novel viruses with zoonotic potential in bats has been described, due to close human–animal contacts [100]. The spillover surveillance is the key for new viral emergence preparedness, for which it is necessary to conduct risk assessments, to monitor wildlife or farms (pigs, poultry, etc.), and to link all these data to human surveillance data. This approach could be useful to set up appropriate containment measures as quickly as possible [101]. In Asia, animal markets are well-known hotspots for viral emergences and, in particular, the bats that are the reservoir for NiV are sold in many street markets [102]. The analysis of NiV genome in bats using PCR and seroprevalence studies can therefore be useful to follow the circulation of strains, including new ones, and to better understand how human outbreaks begin. Indeed, four factors must be present to initiate an epidemic: the transmission intensity in bats, the dynamic of transmission, the shedding of the virus, and the contact between bats and humans via food consumption [30]. Surveillance of all activities that bring animal reservoirs and humans in contact should be implemented in all at-risk countries for NiV, but in the field, there are multiple organizational, financial, logistical, human, and technical challenges. The successful strategy implemented in Vietnam, based on upstream preparedness in the sanitary surveillance of wildlife, domestic animals, and humans, should serve as an example and favor the identification of the areas with the highest risk of emergence [100]. In the state of Kerala in India, a team composed of public health experts, microbiologists, and other infectious disease experts coordinate a multidisciplinary response team to verify the diagnosis, control outbreaks, and identify the source of infection. They collect and identify bats and ensure the quick availability of adequate PPE and other logistics in the area concerned in order to rapidly limit person-to-person transmission in cases of spillover [103]. To be a successful strategy, like in India, this preparedness must be organized before there is a real emergency associated with NiV, by creating concrete collaborations between human health and animal health institutions, thereby making it possible to detect early signals and then develop a more appropriate and faster public health response at the onset of an emergency [103]. The One Health holistic approach applied to NiV thus appears particularly relevant to prevent outbreaks or limit their consequences.

## 7. Discussion

Since 2016, the WHO has added NiV on the Blueprint priority disease list. Thanks to the CEPI efforts, at least 13 vaccine candidates are in the preclinical stages of development, but none of them are licensed, and no therapy is available for this infection. As described above, surveillance activities and the rapid identification of positive cases are still essential to prevent or limit the impact of human outbreaks, and diagnosis is a key factor for this success. In endemic countries, the diagnosis of NiV is still hampered by the few BSL4 or BSL3 laboratories available to safely manage suspicious samples, which means additional time is often required to transport the samples to these facilities. Classical diagnosis in these facilities used mainly expensive and not so easy-to-use RT-PCR assays, which must be implemented on specific and expensive machines by personnel trained for this purpose. Rapid point-of-care tests that can be used at a patient’s bedside could represent an alternative that do not require the extraction of nucleic acids from the samples. They must be quite easy to use but compatible with biosafety, cheap, and not require to well-trained personnel, rendering the diagnosis more rapid and available in remote areas of these countries. Nevertheless, this kind of assay is often less sensitive than other classical diagnosis assays, limiting their use in diagnoses such as those of RG4 pathogens like Nipah virus. For highly pathogenic viruses, it is fundamental to detect the first cases as soon as possible. It is essential not to miss any positive cases because of a significantly low sensitivity assay because that would have serious consequences. These tests are not yet available for NiV except for some CARDs assays that include a large panel of pathogens and that are described in Section 5.1. They remain expensive, and their sensitivity and specificity must be precisely assessed before being recommended for NiV diagnosis in the field. Nevertheless, once the pathogen responsible for the outbreak is well known, and as it has been observed in the field during the Ebola outbreak in West Africa, rapid tests are very useful to quickly identify some positive cases and allow their isolation as soon as possible to minimize the spread of the virus in the population.

RT-PCR methods therefore remain the technique of choice to diagnose NiV, even though an international standard for NiV is yet to be established, and it is difficult to compare the sensitivity of the different assays. Some RT-PCRs have also been validated only on NiV-M or NiV-B but not on both the strains, impacting their use for diagnosis. Indeed, RT-PCR recommended for diagnosis must recognize different members of the *Henipavirus* genus, in order to not miss the emergence of a new viral strains. Moreover, the sensitivity of molecular tests should still be improved due to the false-negative rate (0.7%) observed by an external quality assessment program in China [104].

However, sensitivity of serological assays in the early phase of infection is too low [72] for use in contact tracing during human outbreaks, and these assays are more often used for surveillance. Serology remains an important tool for the surveillance of viral strain circulation in bats and other animals as long as the assays are interpreted correctly. Cross-reaction with *Henipavirus*-like viruses cannot be excluded, and positive detections should be followed by virological studies to detect NiV shedding in animals [105]. With the same limitations, serology is also used to perform seroprevalence studies in humans. These studies are particularly useful in the field for predicting where the next outbreak may occur and to implement preparedness in advance. Nevertheless, the standardization of these tests is currently lacking, and a precise comparison should be performed before recommending the use of some tests in this context. Finally, recent studies suggest that several bats could act as reservoirs for NiV such as *Rosettus aegypticus, Taphozous longimanus, Taphozous melanopagon, Rhinolophus luctus, Chaerophon plicatus,* and *Macroglossus minimus*, indicating that the list of NiV reservoirs can never be considered as definitive [21]. Reliable serological tests will thus be necessary in the future to evaluate the virus circulation in an area that could be larger than initially described.

## 8. Conclusions

For better NiV surveillance and preparedness, all gaps in diagnostic methods should be filled in the near future. Molecular diagnostics for NiV already includes various tests based on different target genes. It is now crucial to implement comparative studies to assess their sensitivity and specificity in order to make some recommendations for future use. Fewer serological tests are available, but the current lack of standardization prevents them from being recommended for diagnostic use.

## Data Availability

Not applicable.

## References

[1] Shoemaker T., Choi M.J. (2017). Henipaviruses. Centers for Disease Control and Prevention CDC Yellow Book 2020: Health Information for International Travel.

[2] Bruno L., Nappo M.A., Ferrari L., Di Lecce R., Guarnieri C., Cantoni A.M., Corradi A. (2022). Nipah Virus Disease: Epidemiological, Clinical, Diagnostic and Legislative Aspects of This Unpredictable Emerging Zoonosis. Animals.

[3] Kummer S., Kranz D.-C. (2022). Henipaviruses—A Constant Threat to Livestock and Humans. PLoS Negl. Trop. Dis..

[4] Sharma V., Kaushik S., Kumar R., Yadav J.P., Kaushik S. (2019). Emerging Trends of Nipah Virus: A Review. Rev. Med. Virol..

[5] Uchida S., Horie R., Sato H., Kai C., Yoneda M. (2018). Possible Role of the Nipah Virus V Protein in the Regulation of the Interferon Beta Induction by Interacting with UBX Domain-Containing Protein1. Sci. Rep..

[6] Chua K.B., Bellini W.J., Rota P.A., Harcourt B.H., Tamin A., Lam S.K., Ksiazek T.G., Rollin P.E., Zaki S.R., Shieh W.-J. (2000). Nipah Virus: A Recently Emergent Deadly Paramyxovirus. Science.

[7] Guillaume V., Aslan H., Ainouze M., Guerbois M., Fabian Wild T., Buckland R., Langedijk J.P.M. (2006). Evidence of a Potential Receptor-Binding Site on the Nipah Virus G Protein (NiV-G): Identification of Globular Head Residues with a Role in Fusion Promotion and Their Localization on an NiV-G Structural Model. J. Virol..

[8] Bonaparte M.I., Dimitrov A.S., Bossart K.N., Crameri G., Mungall B.A., Bishop K.A., Choudhry V., Dimitrov D.S., Wang L.-F., Eaton B.T. (2005). Ephrin-B2 Ligand Is a Functional Receptor for Hendra Virus and Nipah Virus. Proc. Natl. Acad. Sci. USA.

[9] Negrete O.A., Levroney E.L., Aguilar H.C., Bertolotti-Ciarlet A., Nazarian R., Tajyar S., Lee B. (2005). EphrinB2 Is the Entry Receptor for Nipah Virus, an Emergent Deadly Paramyxovirus. Nature.

[10] Aguilar H.C., Matreyek K.A., Filone C.M., Hashimi S.T., Levroney E.L., Negrete O.A., Bertolotti-Ciarlet A., Choi D.Y., McHardy I., Fulcher J.A. (2006). N-Glycans on Nipah Virus Fusion Protein Protect against Neutralization but Reduce Membrane Fusion and Viral Entry. J. Virol..

[11] Patch J.R., Crameri G., Wang L.-F., Eaton B.T., Broder C.C. (2007). Quantitative Analysis of Nipah Virus Proteins Released as Virus-like Particles Reveals Central Role for the Matrix Protein. Virol. J..

[12] Paton N.I., Leo Y.S., Zaki S.R., Auchus A.P., Lee K.E., Ling A.E., Chew S.K., Ang B., Rollin P.E., Umapathi T. (1999). Outbreak of Nipah-Virus Infection among Abattoir Workers in Singapore. Lancet.

[13] Parashar U.D., Sunn L.M., Ong F., Mounts A.W., Arif M.T., Ksiazek T.G., Kamaluddin M.A., Mustafa A.N., Kaur H., Ding L.M. (2000). Case-Control Study of Risk Factors for Human Infection with a New Zoonotic Paramyxovirus, Nipah Virus, during a 1998–1999 Outbreak of Severe Encephalitis in Malaysia. J. Infect. Dis..

[14] Rahman S.A., Hassan S.S., Olival K.J., Mohamed M., Chang L.-Y., Hassan L., Saad N.M., Shohaimi S.A., Mamat Z.C., Naim M.S. (2010). Characterization of Nipah Virus from Naturally Infected Pteropus vampyrus Bats, Malaysia. Emerg. Infect. Dis..

[15] Anderson D.E., Islam A., Crameri G., Todd S., Islam A., Khan S.U., Foord A., Rahman M.Z., Mendenhall I.H., Luby S.P. (2019). Isolation and Full-Genome Characterization of Nipah Viruses from Bats, Bangladesh. Emerg. Infect. Dis..

[16] Yob J.M., Field H., Rashdi A.M., Morrissy C., Van Der Heide B., Rota P., Bin Adzhar A., White J., Daniels P., Jamaluddin A. (2001). Nipah Virus Infection in Bats (Order Chiroptera) in Peninsular Malaysia. Emerg. Infect. Dis..

[17] Yadav P.D., Towner J.S., Raut C.G., Shete A.M., Nichol S.T., Mourya D.T., Mishra A.C. (2012). Detection of Nipah Virus RNA in Fruit Bat (Pteropus giganteus) from India. Am. J. Trop. Med. Hyg..

[18] Halpin K., Hyatt A.D., Fogarty R., Middleton D., Bingham J., Epstein J.H., Rahman S.A., Hughes T., Smith C., Field H.E. (2011). Pteropid Bats Are Confirmed as the Reservoir Hosts of Henipaviruses: A Comprehensive Experimental Study of Virus Transmission. Am. J. Trop. Med. Hyg..

[19] Chow V.T.K., Tambyah P.A., Yeo W.M., Phoon M.C., Howe J. (2000). Diagnosis of Nipah Virus Encephalitis by Electron Microscopy of Cerebrospinal Fluid. J. Clin. Virol..

[20] Ambat A.S., Zubair S.M., Prasad N., Pundir P., Rajwar E., Patil D.S., Mangad P. (2019). Nipah Virus: A Review on Epidemiological Characteristics and Outbreaks to Inform Public Health Decision Making. J. Infect. Public Health.

[21] Plowright R.K., Becker D.J., Crowley D.E., Washburne A.D., Huang T., Nameer P.O., Gurley E.S., Han B.A. (2019). Prioritizing Surveillance of Nipah Virus in India. PLoS Negl. Trop. Dis..

[22] Yadav P.D., Shete A.M., Kumar G.A., Sarkale P., Sahay R.R., Radhakrishnan C., Lakra R., Pardeshi P., Gupta N., Gangakhedkar R.R. (2019). Nipah Virus Sequences from Humans and Bats during Nipah Outbreak, Kerala, India, 2018. Emerg. Infect. Dis..

[23] Mhod Nor M.N., Gan C.H., Ong B.L. (2000). Nipah Virus Infection of Pigs in Peninsular Malaysia. Rev. Sci. Tech. OIE.

[24] Chua K.B. (2003). Nipah Virus Outbreak in Malaysia. J. Clin. Virol..

[25] Enserink M. (1999). New Virus Fingered in Malaysian Epidemic. Science.

[26] Centers for Disease Control and Prevention (CDC) (1999). Update: Outbreak of Nipah Virus—Malaysia and Singapore, 1999. MMWR Morb. Mortal. Wkly. Rep..

[27] Chowdhury S., Khan S.U., Crameri G., Epstein J.H., Broder C.C., Islam A., Peel A.J., Barr J., Daszak P., Wang L.-F. (2014). Serological Evidence of Henipavirus Exposure in Cattle, Goats and Pigs in Bangladesh. PLoS Negl. Trop. Dis..

[28] Hegde S.T., Sazzad H.M.S., Hossain M.J., Alam M.-U., Kenah E., Daszak P., Rollin P., Rahman M., Luby S.P., Gurley E.S. (2016). Investigating Rare Risk Factors for Nipah Virus in Bangladesh: 2001–2012. EcoHealth.

[29] Rahman M., Chakraborty A. (2012). Nipah Virus Outbreaks in Bangladesh: A Deadly Infectious Disease. WHO South-East Asia J. Public Health.

[30] Epstein J.H., Anthony S.J., Islam A., Kilpatrick A.M., Ali Khan S., Balkey M.D., Ross N., Smith I., Zambrana-Torrelio C., Tao Y. (2020). Nipah Virus Dynamics in Bats and Implications for Spillover to Humans. Proc. Natl. Acad. Sci. USA.

[31] Ching P.K.G., De Los Reyes V.C., Sucaldito M.N., Tayag E., Columna-Vingno A.B., Malbas F.F., Bolo G.C., Sejvar J.J., Eagles D., Playford G. (2015). Outbreak of Henipavirus Infection, Philippines, 2014. Emerg. Infect. Dis..

[32] Chua K.B., Goh K.J., Wong K.T., Kamarulzaman A., Tan P.S.K., Ksiazek T.G., Zaki S.R., Paul G., Lam S.K., Tan C.T. (1999). Fatal Encephalitis Due to Nipah Virus among Pig-Farmers in Malaysia. Lancet.

[33] Gurley E.S., Spiropoulou C.F., De Wit E. (2020). Twenty Years of Nipah Virus Research: Where Do We Go From Here?. J. Infect. Dis..

[34] Arankalle V.A., Bandyopadhyay B.T., Ramdasi A.Y., Jadi R., Patil D.R., Rahman M., Majumdar M., Banerjee P.S., Hati A.K., Goswami R.P. (2011). Genomic Characterization of Nipah Virus, West Bengal, India. Emerg. Infect. Dis..

[35] Kumar C.P.G., Sugunan A.P., Yadav P., Kurup K.K., Aarathee R., Manickam P., Bhatnagar T., Radhakrishnan C., Thomas B., Kumar A. (2019). Infections among Contacts of Patients with Nipah Virus, India. Emerg. Infect. Dis..

[36] Hossain M.J., Gurley E.S., Montgomery J.M., Bell M., Carroll D.S., Hsu V.P., Formenty P., Croisier A., Bertherat E., Faiz M.A. (2008). Clinical Presentation of Nipah Virus Infection in Bangladesh. Clin. Infect. Dis..

[37] Playford E.G., McCall B., Smith G., Slinko V., Allen G., Smith I., Moore F., Taylor C., Kung Y.-H., Field H. (2010). Human Hendra Virus Encephalitis Associated with Equine Outbreak, Australia, 2008. Emerg. Infect. Dis..

[38] Arunkumar G., Chandni R., Mourya D.T., Singh S.K., Sadanandan R., Sudan P., Bhargava B., Gangakhedkar R.R., Gupta N., Nipah Investigators People and Health Study Group (2019). Outbreak Investigation of Nipah Virus Disease in Kerala, India, 2018. J. Infect. Dis..

[39] Goh K.J., Tan C.T., Chew N.K., Tan P.S.K., Kamarulzaman A., Sarji S.A., Wong K.T., Abdullah B.J.J., Chua K.B., Lam S.K. (2000). Clinical Features of Nipah Virus Encephalitis among Pig Farmers in Malaysia. N. Engl. J. Med..

[40] Aditi, Shariff M. (2019). Aditi; Shariff, M. Nipah Virus Infection: A Review. Epidemiol. Infect..

[41] World Health Organization Nipah Virus. https://www.who.int/news-room/fact-sheets/detail/nipah-virus.

[42] Hauser N., Gushiken A.C., Narayanan S., Kottilil S., Chua J.V. (2021). Evolution of Nipah Virus Infection: Past, Present, and Future Considerations. Trop. Med. Infect. Dis..

[43] Ang B.S.P., Lim T.C.C., Wang L. (2018). Nipah Virus Infection. J. Clin. Microbiol..

[44] Pallivalappil B., Ali A., Thulaseedharan N., Karadan U., Chellenton J., Dipu K., Anoop Kumar A., Sajeeth Kumar K., Rajagopal T., Suraj K. (2020). Dissecting an Outbreak: A Clinico-Epidemiological Study of Nipah Virus Infection in Kerala, India, 2018. J. Glob. Infect. Dis..

[45] Wong K.T., Shieh W.-J., Kumar S., Norain K., Abdullah W., Guarner J., Goldsmith C.S., Chua K.B., Lam S.K., Tan C.T. (2002). Nipah Virus Infection: Pathology and Pathogenesis of an Emerging Paramyxoviral Zoonosis. Am. J. Pathol..

[46] Singh R.K., Dhama K., Chakraborty S., Tiwari R., Natesan S., Khandia R., Munjal A., Vora K.S., Latheef S.K., Karthik K. (2019). Nipah Virus: Epidemiology, Pathology, Immunobiology and Advances in Diagnosis, Vaccine Designing and Control Strategies—A Comprehensive Review. Vet. Q..

[47] Siva S.R., Chong H.T., Tan C.T. (2009). Ten Year Clinical and Serological Outcomes of Nipah Virus Infection. Neurol. Asia.

[48] Sejvar J.J., Hossain J., Saha S.K., Gurley E.S., Banu S., Hamadani J.D., Faiz M.A., Siddiqui F.M., Mohammad Q.D., Mollah A.H. (2007). Long-Term Neurological and Functional Outcome in Nipah Virus Infection. Ann. Neurol..

[49] Thakur N., Bailey D. (2019). Advances in Diagnostics, Vaccines and Therapeutics for Nipah Virus. Microbes Infect..

[50] Tan K.S., Tan C.T., Goh K.J. (1999). Epidemiological Aspects of Nipah Virus Infection. Neurol. J. Southeast Asia.

[51] Chan K.P., Rollin P.E., Ksiazek T.G., Leo Y.S., Goh K.T., Paton N.I., Sng E.H., Ling A.E. (2002). A Survey of Nipah Virus Infection among Various Risk Groups in Singapore. Epidemiol. Infect..

[52] Hsu V.P., Hossain M.J., Parashar U.D., Ali M.M., Ksiazek T.G., Kuzmin I., Niezgoda M., Rupprecht C., Bresee J., Breiman R.F. (2004). Nipah Virus Encephalitis Reemergence, Bangladesh. Emerg. Infect. Dis..

[53] Banerjee S., Gupta N., Kodan P., Mittal A., Ray Y., Nischal N., Soneja M., Biswas A., Wig N. (2019). Nipah Virus Disease: A Rare and Intractable Disease. Intractable Rare Dis. Res..

[54] Wong K.T., Robertson T., Ong B.B., Chong J.W., Yaiw K.C., Wang L.F., Ansford A.J., Tannenberg A. (2009). Human Hendra Virus Infection Causes Acute and Relapsing Encephalitis. Neuropathol. Appl. Neurobiol..

[55] Chong H.T., Kunjapan S.R., Thayaparan T., Geok Tong J.M., Petharunam V., Jusoh M.R., Tan C.T. (2002). Nipah Encephalitis Outbreak in Malaysia, Clinical Features in Patients from Seremban. Can. J. Neurol. Sci..

[56] Middleton D.J., Westbury H.A., Morrissy C.J., Van Der Heide B.M., Russell G.M., Braun M.A., Hyatt A.D. (2002). Experimental Nipah Virus Infection in Pigs and Cats. J. Comp. Pathol..

[57] Centers for Disease Control and Prevention (CDC) Nipah Virus (NiV). https://www.cdc.gov/vhf/nipah/index.html.

[58] Centers for Disease Control and Prevention Nipah Virus (NiV): Diagnosis. https://www.cdc.gov/vhf/nipah/diagnosis/index.html.

[59] Tong S., Chern S.-W.W., Li Y., Pallansch M.A., Anderson L.J. (2008). Sensitive and Broadly Reactive Reverse Transcription-PCR Assays To Detect Novel Paramyxoviruses. J. Clin. Microbiol..

[60] Wacharapluesadee S., Lumlertdacha B., Boongird K., Wanghongsa S., Chanhome L., Rollin P., Stockton P., Rupprecht C.E., Ksiazek T.G., Hemachudha T. (2005). Bat Nipah Virus, Thailand. Emerg. Infect. Dis..

[61] Wacharapluesadee S., Boongird K., Wanghongsa S., Phumesin P., Hemachudha T. (2006). Drinking Bat Blood May Be Hazardous to Your Health. Clin. Infect. Dis..

[62] Guillaume V., Lefeuvre A., Faure C., Marianneau P., Buckland R., Lam S.K., Wild T.F., Deubel V. (2004). Specific Detection of Nipah Virus Using Real-Time RT-PCR (TaqMan). J. Virol. Methods.

[63] Feldman K.S., Foord A., Heine H.G., Smith I.L., Boyd V., Marsh G.A., Wood J.L.N., Cunningham A.A., Wang L.-F. (2009). Design and Evaluation of Consensus PCR Assays for Henipaviruses. J. Virol. Methods.

[64] Chang L.-Y., Mohd Ali A., Hassan S.S., AbuBakar S. (2006). Quantitative Estimation of Nipah Virus Replication Kinetics in Vitro. Virol. J..

[65] Jensen K.S., Adams R., Bennett R.S., Bernbaum J., Jahrling P.B., Holbrook M.R. (2018). Development of a Novel Real-Time Polymerase Chain Reaction Assay for the Quantitative Detection of Nipah Virus Replicative Viral RNA. PLoS ONE.

[66] Onyango C.O., Loparev V., Lidechi S., Bhullar V., Schmid D.S., Radford K., Lo M.K., Rota P., Johnson B.W., Munoz J. (2017). Evaluation of a TaqMan Array Card for Detection of Central Nervous System Infections. J. Clin. Microbiol..

[67] Liu J., Ochieng C., Wiersma S., Ströher U., Towner J.S., Whitmer S., Nichol S.T., Moore C.C., Kersh G.J., Kato C. (2016). Development of a TaqMan Array Card for Acute-Febrile-Illness Outbreak Investigation and Surveillance of Emerging Pathogens, Including Ebola Virus. J. Clin. Microbiol..

[68] Ma L., Chen Z., Guan W., Chen Q., Liu D. (2019). Rapid and Specific Detection of All Known Nipah Virus Strains’ Sequences With Reverse Transcription-Loop-Mediated Isothermal Amplification. Front. Microbiol..

[69] Pollak N.M., Olsson M., Marsh G.A., Macdonald J., McMillan D. (2023). Evaluation of Three Rapid Low-Resource Molecular Tests for Nipah Virus. Front. Microbiol..

[70] Wylezich C., Calvelage S., Schlottau K., Ziegler U., Pohlmann A., Höper D., Beer M. (2021). Next-Generation Diagnostics: Virus Capture Facilitates a Sensitive Viral Diagnosis for Epizootic and Zoonotic Pathogens Including SARS-CoV-2. Microbiome.

[71] Arunkumar G., Devadiga S., McElroy A.K., Prabhu S., Sheik S., Abdulmajeed J., Robin S., Sushama A., Jayaram A., Nittur S. (2019). Adaptive Immune Responses in Humans During Nipah Virus Acute and Convalescent Phases of Infection. Clin. Infect. Dis..

[72] Ramasundram V., Tan C.T., Chua K.B., Chong H.T., Goh K.J., Chew N.K., Tan K.S., Thayaparan T., Kunjapan S.R., Petharunam V. (2000). Kinetics of IgM and IgG Seroconversion in Nipah Virus Infection. Neurol. J. Southeast Asia.

[73] Shete A., Radhakrishnan C., Pardeshi P., Yadav P., Jain R., Sahay R., Sugunan A. (2021). Antibody Response in Symptomatic & Asymptomatic Nipah Virus Cases from Kerala, India. Indian J. Med. Res..

[74] Kulkarni D.D., Tosh C., Venkatesh G., Senthil Kumar D. (2013). Nipah Virus Infection: Current Scenario. Indian J. Virol..

[75] Abdullah S., Tan C.T. (2014). Henipavirus Encephalitis. Handbook of Clinical Neurology.

[76] Satterfield B.A., Dawes B.E., Milligan G.N. (2016). Status of Vaccine Research and Development of Vaccines for Nipah Virus. Vaccine.

[77] Chattu V., Kumar R., Kumary S., Kajal F., David J. (2018). Nipah Virus Epidemic in Southern India and Emphasizing “One Health” Approach to Ensure Global Health Security. J. Fam. Med. Prim. Care.

[78] Daniels P., Ksiazek T., Eaton B.T. (2001). Laboratory Diagnosis of Nipah and Hendra Virus Infections. Microbes Infect..

[79] Field H., Young P., Yob J.M., Mills J., Hall L., Mackenzie J. (2001). The Natural History of Hendra and Nipah Viruses. Microbes Infect..

[80] Harcourt B.H., Tamin A., Ksiazek T.G., Rollin P.E., Anderson L.J., Bellini W.J., Rota P.A. (2000). Molecular Characterization of Nipah Virus, a Newly Emergent Paramyxovirus. Virology.

[81] Yu F., Khairullah N.S., Inoue S., Balasubramaniam V., Berendam S.J., Teh L.K., Ibrahim N.S.W., Abdul Rahman S., Hassan S.S., Hasebe F. (2006). Serodiagnosis Using Recombinant Nipah Virus Nucleocapsid Protein Expressed in Escherichia coli. J. Clin. Microbiol..

[82] Kulkarni D.D., Venkatesh G., Tosh C., Patel P., Mashoria A., Gupta V., Gupta S., Senthilkumar D. (2016). Development and Evaluation of Recombinant Nucleocapsid Protein Based Diagnostic ELISA for Detection of Nipah Virus Infection in Pigs. J. Immunoassay Immunochem..

[83] Chiang C.-F., Lo M.K., Rota P.A., Spiropoulou C.F., Rollin P.E. (2010). Use of Monoclonal Antibodies against Hendra and Nipah Viruses in an Antigen Capture ELISA. Virol. J..

[84] Ksiazek T.G., Rollin P.E., Jahrling P.B., Johnson E., Dalgard D.W., Peters C.J. (1992). Enzyme Immunosorbent Assay for Ebola Virus Antigens in Tissues of Infected Primates. J. Clin. Microbiol..

[85] Saijo M., Georges-Courbot M.-C., Fukushi S., Mizutani T., Philippe M., Georges A.-J., Kurane I., Morikawa S. (2006). Marburgvirus Nucleoprotein-Capture Enzyme-Linked Immunosorbent Assay Using Monoclonal Antibodies to Recombinant Nucleoprotein: Detection of Authentic Marburgvirus. Jpn. J. Infect. Dis..

[86] Warnes A., Fooks A.R., Stephenson J.R. (2004). Design and Preparation of Recombinant Antigens as Diagnostic Reagents in Solid-Phase Immunosorbent Assays. Methods Mol. Med..

[87] Chen J.-M., Yu M., Morrissy C., Zhao Y.-G., Meehan G., Sun Y.-X., Wang Q.-H., Zhang W., Wang L.-F., Wang Z.-L. (2006). A Comparative Indirect ELISA for the Detection of Henipavirus Antibodies Based on a Recombinant Nucleocapsid Protein Expressed in Escherichia Coli. J. Virol. Methods.

[88] Kashiwazaki Y., Na Y.N., Tanimura N., Imada T. (2004). A Solid-Phase Blocking ELISA for Detection of Antibodies to Nipah Virus. J. Virol. Methods.

[89] McNabb L., Barr J., Crameri G., Juzva S., Riddell S., Colling A., Boyd V., Broder C., Wang L.-F., Lunt R. (2014). Henipavirus Microsphere Immuno-Assays for Detection of Antibodies against Hendra Virus. J. Virol. Methods.

[90] Foord A.J., White J.R., Colling A., Heine H.G. (2013). Microsphere Suspension Array Assays for Detection and Differentiation of Hendra and Nipah Viruses. BioMed Res. Int..

[91] Wang L.-F., Daniels P., Lee B., Rota P.A. (2012). Diagnosis of Henipavirus Infection: Current Capabilities and Future Directions. Henipavirus.

[92] Bae S.E., Kim S.S., Moon S.T., Cho Y.D., Lee H., Lee J.-Y., Shin H.Y., Lee H.-J., Kim Y.B. (2019). Construction of the Safe Neutralizing Assay System Using Pseudotyped Nipah Virus and G Protein-Specific Monoclonal Antibody. Biochem. Biophys. Res. Commun..

[93] Kaku Y., Noguchi A., Marsh G.A., McEachern J.A., Okutani A., Hotta K., Bazartseren B., Fukushi S., Broder C.C., Yamada A. (2009). A Neutralization Test for Specific Detection of Nipah Virus Antibodies Using Pseudotyped Vesicular Stomatitis Virus Expressing Green Fluorescent Protein. J. Virol. Methods.

[94] Khetawat D., Broder C.C. (2010). A Functional Henipavirus Envelope Glycoprotein Pseudotyped Lentivirus Assay System. Virol. J..

[95] Kaku Y., Noguchi A., Marsh G.A., Barr J.A., Okutani A., Hotta K., Bazartseren B., Fukushi S., Broder C.C., Yamada A. (2012). Second Generation of Pseudotype-Based Serum Neutralization Assay for Nipah Virus Antibodies: Sensitive and High-Throughput Analysis Utilizing Secreted Alkaline Phosphatase. J. Virol. Methods.

[96] Tamin A., Harcourt B.H., Lo M.K., Roth J.A., Wolf M.C., Lee B., Weingartl H., Audonnet J.-C., Bellini W.J., Rota P.A. (2009). Development of a Neutralization Assay for Nipah Virus Using Pseudotype Particles. J. Virol. Methods.

[97] Hyatt A.D., Zaki S.R., Goldsmith C.S., Wise T.G., Hengstberger S.G. (2001). Ultrastructure of Hendra Virus and Nipah Virus within Cultured Cells and Host Animals. Microbes Infect..

[98] Chua K.B., Lam S.K., Tan C.T., Hooi P.S., Goh K.J., Chew N.K., Tan K.S., Kamarulzaman A., Wong K.T. (2000). High Mortality in Nipah Encephalitis Is Associated with Presence of Virus in Cerebrospinal Fluid. Ann. Neurol..

[99] World Health Organization One Health. https://www.who.int/health-topics/one-health#tab=tab_1.

[100] Latinne A., Nga N.T.T., Long N.V., Ngoc P.T.B., Thuy H.B., Long N.V., Long P.T., Phuong N.T., Quang L.T.V., PREDICT Consortium (2023). One Health Surveillance Highlights Circulation of Viruses with Zoonotic Potential in Bats, Pigs, and Humans in Viet Nam. Viruses.

[101] Charlier J., Barkema H.W., Becher P., De Benedictis P., Hansson I., Hennig-Pauka I., La Ragione R., Larsen L.E., Madoroba E., Maes D. (2022). Disease Control Tools to Secure Animal and Public Health in a Densely Populated World. Lancet Planet. Health.

[102] Morcatty T.Q., Pereyra P.E.R., Ardiansyah A., Imron M.A., Hedger K., Campera M., Nekaris K.A.-I., Nijman V. (2022). Risk of Viral Infectious Diseases from Live Bats, Primates, Rodents and Carnivores for Sale in Indonesian Wildlife Markets. Viruses.

[103] Singhai M., Jain R., Jain S., Bala M., Singh S., Goyal R. (2021). Nipah Virus Disease: Recent Perspective and One Health Approach. Ann. Glob. Health.

[104] Zhang R., Tan P., Feng L., Li R., Yang J., Zhang R., Li J. (2021). External Quality Assessment of Molecular Testing of 9 Viral Encephalitis-Related Viruses in China. Virus Res..

[105] Gilbert A.T., Fooks A.R., Hayman D.T.S., Horton D.L., Müller T., Plowright R., Peel A.J., Bowen R., Wood J.L.N., Mills J. (2013). Deciphering Serology to Understand the Ecology of Infectious Diseases in Wildlife. EcoHealth.

